# The Backbones of the Poor

**Published:** 1888-03-31

**Authors:** 


### The Doctor's Pulpit.

### THE BACKBONES OF THE POOR.

A Radical contemporary affirms that in these days "philanthropy thinks much of smoothing down the surface of sorrow," but "little of building up character." The writer does not take the trouble to say what he means by "philanthropy." According to the religious world, the building up of character is a special branch of philanthropy, and the most important of all branches. Money spent on home and foreign missions, the distribution of the Scriptures, and other evangelistic purposes, is believed by the donors to be devoted entirely to the moral improvement of men, and is given solely for that purpose. The *Pall Mall Gazette* seems to be of opinion that the philanthropy which relieves poverty and builds hospitals should also aim at "building up the character."

There is a wide-spread belief that the character of the classes who need hospital and other relief stands in much need of building up. According to some, who think they know, it is in a very shaky and tumble-down condition. There are those who affirm that in many cases it is little better than a ruin, and is so rapidly going from bad to worse that very soon there will be no possibility of building it up at all.

We are not prepared to deny these charges. It is possible, however, that they apply with equal force to large numbers of the middle and upper classes. But we are concerned now with those who do not lay up stores against a rainy day, and who therefore, as soon as work fails or illness makes its appearance, must go cap in hand to the nearest hospital, or seek some other form of charitable relief.

Is it true that the working-classes of towns have no moral backbones? That is really the gist of the whole matter. Are they quite satisfied to live from day to day without attempting to make provision for the future? Are they perfectly content when ill to go to the out-patient department of the hospital, or to seek admittance within its walls, and to leave their families to be fed by the ravens, like the prophet Elijah, or not to be fed at all, if the ravens, as is more than probable, should fail to make their appearance? In other words, is it true that the working population is a class by itself, totally distinct and different from other classes, and destitute of the sober and resolute purposes and worthy ambitions of other classes? Does that class consist, as is sometimes said, of "the residuum, and those who are gradually settling down towards the residuum"?

In the animal world there are plenty of creatures which have no backbones; but they are every one, without exception, of a lower organisation than those which have backbones. At the very bottom of the scale are creatures which have not the least pretence to backbones. These are not merely backboneless, but boneless in every other respect, and as destitute of brain as of bone. What are they made for? To be eaten chiefly, and eaten they surely are. The fact is a parable. It is quite in accordance with natural science that lower organisms are eaten by higher, throughout the whole scale of animal existence. But the law is not confined to animal existence. It is just as operative in the mental and moral spheres. Does the sheep make a dinner for the wolf? Just so does the man of sheepish character make a meal for the man of wolfish predilections. Does the lion eat up the calf? As surely does the human lion devour the man whose brains and courage are of the calf type. The fact is certain and inevitable, explain it as we may, and exclaim against it how we will.

It seems as if the Maker and Ruler of men were determined that they should be creatures of intelligence, independent judgment, and resolute courage. So definite and assured are the high rewards which fall to those who display these characteristics, and so wretched and miserable are the penalties which fall upon those who fail of them, that the conclusion is unavoidable to the reasoning mind. As a rule, there is the strictest possible proportion between the degree of failure from what may be considered the natural standard of intelligence and character for a man, and the nature and severity of the penalties which follow such failure. If a man have pluck, but not much intelligence, he will have one kind of success: if he have intelligence, but not courage, he will have another kind. If he have capacity without conscience, he will reach one elevation; if he have conscience without capacity, he will reach quite a different standing-ground. Although it is quite true that the race is not *always* to the swift, nor the battle *always* to the strong, but that time and chance happen to them all; yet it is equally true that the battle is *generally* to the strong, and the race *generally* to the swift; and that is exactly what Solomon meant to say. In saying that *sometimes* the swift failed to win the race, he merely desired to point out that there were exceptions to every rule.

This journal is devoted to philanthropy among other things; but it aims at philanthropy in the widest possible sense. Charitable relief is good and necessary in many cases, and should never be withheld or grudgingly supplied to those who are in actual need. Religion and science are quite at one in believing that it is wasteful as well as wicked to let men and women lack food when hungry, or medical skill when sick. But they are equally agreed that though it is good and wise to feed the hungry and give medical aid to the sick, it is infinitely better, when possible, for the hungry to find food for himself, and for the sick to provide his own medical relief. It is necessary now, and it may be necessary always, to give charitable relief to some; but a wise philanthropy will constantly strive to lessen the number of those needing such relief, and to increase the proportion of those who are resolved to provide for all their own wants. The first step in the direction of such self-help is for the working classes to have the desire and the determination to provide for themselves, like other classes.

We suppose ourselves to be addressing many of those who live by the work of their hands. We venture to recall to their minds what we have said about low organisms in the animal world. So far as appears, *they are made to be devoured.* We repeat our conviction—a conviction amply justified by the world's six thousand years' experience—that there is a strict parallelism between that which happens to low physical organisms and that which is the lot of human beings of low mental aptitudes. The African has a feebler courage and a less resolute mind than the Arab, and the Arab drives him in hundreds to the slave market. The Red Indian has less intelligence and a weaker purpose than the white man; and he has everywhere gone down before him, almost to the point of extinction. It is exactly the same everywhere. If working men have not the courage, energy, and foresight to look ahead like other men, and to provide against possible contingencies, they are, and always must be, entirely at the mercy of those immediately above them. If they cannot hold their own and go forward, they must go more and more backward and downward, until they are left without weapons of any kind in the daily conflict. If they will not stand up for themselves, look forward for themselves, think for themselves, work for themselves, and fight for themselves, it is impossible for any other class to maintain them in their natural position of independent manhood. Creatures without backbones are made to be eaten by creatures with backbones, and are almost invariably so eaten. If men of any rank shuffle themselves out of those mental and moral backbones which Nature has provided them with, they will be devoured too.

